# Difficulty Regulating Social Media Content of Age-Restricted Products: Comparing JUUL’s Official Twitter Timeline and Social Media Content About JUUL

**DOI:** 10.2196/29011

**Published:** 2021-12-07

**Authors:** Danny Valdez, Jennifer B Unger

**Affiliations:** 1 Department of Applied Health Science Indiana University School of Public Health Bloomington, IN United States; 2 Keck School of Medicine University of Southern California Los Angeles, CA United States

**Keywords:** social media, JUUL, underage marketing, LDA, Latent Dirichlet Allocation, topic models

## Abstract

**Background:**

In 2018, JUUL Labs Inc, a popular e-cigarette manufacturer, announced it would substantially limit its social media presence in compliance with the Food and Drug Administration’s (FDA) call to curb underage e-cigarette use. However, shortly after the announcement, a series of JUUL-related hashtags emerged on various social media platforms, calling the effectiveness of the FDA’s regulations into question.

**Objective:**

The purpose of this study is to determine whether hashtags remain a common venue to market age-restricted products on social media.

**Methods:**

We used Twitter’s standard application programming interface to download the 3200 most-recent tweets originating from JUUL Labs Inc’s official Twitter Account (@JUULVapor), and a series of tweets (n=28,989) from other Twitter users containing either #JUUL or mentioned JUUL in the tweet text. We ran exploratory (10×10) and iterative Latent Dirichlet Allocation (LDA) topic models to compare @JUULVapor’s content versus our hashtag corpus. We qualitatively deliberated topic meanings and substantiated our interpretations with tweets from either corpus.

**Results:**

The topic models generated for @JUULVapor’s timeline seemingly alluded to compliance with the FDA’s call to prohibit marketing of age-restricted products on social media. However, the topic models generated for the hashtag corpus of tweets from other Twitter users contained several references to flavors, vaping paraphernalia, and illicit drugs, which may be appealing to younger audiences.

**Conclusions:**

Our findings underscore the complicated nature of social media regulation. Although JUUL Labs Inc seemingly complied with the FDA to limit its social media presence, JUUL and other e-cigarette manufacturers are still discussed openly in social media spaces. Much discourse about JUUL and e-cigarettes is spread via hashtags, which allow messages to reach a wide audience quickly. This suggests that social media regulations on manufacturers cannot prevent e-cigarette users, influencers, or marketers from spreading information about e-cigarette attributes that appeal to the youth, such as flavors. Stricter protocols are needed to regulate discourse about age-restricted products on social media.

## Introduction

Following the Food and Drug Administration’s (FDA’s) call to curb underage e-cigarette use and increasing criticism of JUUL’s youth-oriented “Vaporized” campaign [1], JUUL Labs Inc announced it would limit its social media presence. As part of the FDA agreement, JUUL deleted its official Facebook and Instagram accounts, reduced its Twitter activity, and removed older Twitter posts that could be attractive to youth or interpreted as marketing to youth [1].

FDA can regulate what JUUL and other e-cigarette manufacturers can post on official social media platforms [2]. However, the FDA cannot regulate posts about JUUL by customers or influencers, who can identify their posts as JUUL-related by using hashtags—short words or phrases preceded by the ‘#’ symbol that label the content of a social media post and cause the post to appear in users’ keyword searches. Hashtags spread social media content rapidly [3] and are therefore used for branding and marketing of certain products for mainstream appeal [4]. Any social media user (including paid or unpaid social media influencers, retailers, or enthusiastic consumers) can use hashtags to spread content about any topic, including age-restricted products subject to federal regulations. Alcohol, for example, is heavily marketed through hashtags [5-7], though much social media content about alcohol does not originate from official corporate accounts.

Marketing research further suggests that hashtags are used as branding or marketing ploys to promote age-restricted products including alcohol [7] and tobacco [8] on social networking websites. Using hashtags for age-restricted products may help circumvent age-gates, which are already proven to be ineffective at deterring underage engagement with age-restricted products [9]. Thus, any effort by JUUL Labs Inc to curb marketing to underage users may be stunted by the presence (and popularity) of vaping-related hashtags not subject to regulations imposed on manufacturers.

Indeed, the prevalence of JUUL-related hashtags on Instagram increased after JUUL reduced its own social media presence [1]. This suggests a limitation to FDA regulations wherein age-restricted products can still be marketed separately from official company platforms. By consequence, age-restricted items that are popular among youth including alcohol, tobacco, and e-cigarettes remain overtly visible and marketable to this audience, despite official corporate positions that denounce such use.

Regulation of harmful social media content is a critical public health issue [10]. To our knowledge, however, no study has compared verified corporate accounts versus similarly related hashtags from noncorporate posters to examine the effectiveness of social media regulation efforts. This study uses an inductive approach and natural language processing (NLP) modeling to examine differences in JUUL’s official, regulated Twitter account @JUULVapor, JUUL-related content posted on social media. Our study is guided by three research questions:

1. Do people’s social media posts about JUUL provide evidence of greater reach and visibility of JUUL Labs Inc’s official Twitter account @JUULVapor?
2. Can we leverage Latent Dirichlet Allocation (LDA) topic models to dissect JUUL-related corpora?
3. What are salient content differences between @JUULVapor and tweets published by social media users containing either #JUUL or “JUUL” within the tweet’s text?

Collectively, findings from our study will contribute to discourse about information diffusion via social media. We hypothesize that despite JUUL’s efforts to scrub their social media platforms of youth-oriented content, hashtags about JUUL remain pervasive and highly visible to youth.

## Methods

### Data

Data for this study were procured by leveraging Twitter’s application programming interface (API). From the API, we collected two corpora unique to this study: (1) @JUULVapor’s Twitter timeline (n=3200 tweets, the maximum number of most recent tweets posted by a single user allotted for download through the standard API), hereafter referred to as the @JUULVapor corpus (January 1 to May 31, 2021) and (2) a 1-month collection of tweets containing #JUUL or “JUUL” (n=29,989 tweets), referred to as the #JUUL corpus (May 1 to June 1, 2021). For the #JUUL corpus, specifically, we performed a Bot analysis [11] to remove tweets that originated from nonhuman accounts (n=135). No Bot analysis was required for the @JUULVapor corpus considering those tweets were pulled from JUUL Lab Inc’s official Twitter account. We performed this procedure to ensure that discourse captured in subsequent analyses originated from humans and not an automated program. Upon removing bot accounts, 2 raters independently reviewed the text of the #JUUL tweets and removed any from the corpus that were not expressly about e-cigarettes or vaping (n=23). An author of this study also cross-checked tweet IDs in either corpus to ensure there was no accidental overlap in tweets (ie, the same tweet appearing in both the @JUUL and #JUUL corpora). Note that our total sample inclusive of both corpora (N=33,189 tweets) exceeds the mean observed sample size of collected tweets in a meta-analysis of public health social media studies (n=10,000) [12].

### Analysis

Our research questions are exploratory. Thus, we chose to use LDA topic models, a Bayes-driven, unsupervised NLP method, to examine differences in themes for the @JUULVapor and #JUUL corpora. LDA and related topic modeling analyses have been similarly leveraged in other health contexts, including studying discourse about the COVID-19 pandemic [13] and map themes among corpora of age-restricted products [14].

While previous studies generate topic models for differing corpora, qualitatively review differences between corpora, and discuss the meaning of those differences, our study takes a 2-step approach. The first step broadly examines themes for a fixed set of topics or words per topic (ie, 10 topics and 10 words per topic). Valdez et al [15] have provided examples of exploratory topic models in practice. The second step uses an iterative topic model analysis that meta-analytically generates models with an increasing number of topics per corpus (ie, 1 topic, 2 topics…20 topics) [16]. This analysis generates a coherence score for each iteration, such that higher scores are equated with better model fit and interpretability. We used this second analysis to identify the optimal number of topics per corpus and further refine the models (ie, eliminate redundancy and noise) for maximum interpretability. To ensure the validity of our coherence scores, we selected a random sample of 50 tweets per corpus and matched each tweet’s content to a respective theme identified by the topic model. We successfully placed each tweet within a topic, suggesting our topic models were both coherent and precise.

### Procedure

**Figure 1:**
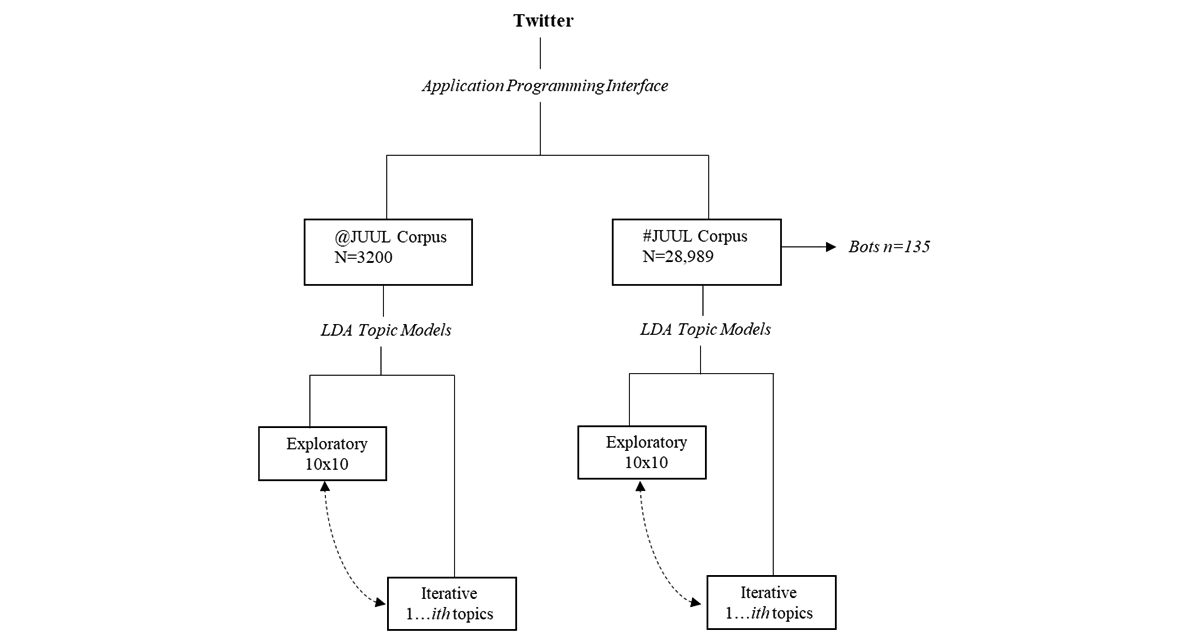
Conceptual framework guiding our study. LDA: Latent Dirichlet Allocation.

Our workflow is detailed in Figure 1. Upon downloading and cleaning the @JUULVapor and #JUUL corpora, we performed the following. First, we calculated standard descriptive statistics for each corpus, including the average number of likes, retweets, and number of tweets that originated from Verified accounts, or accounts reviewed to ensure they are owned and operated by a specific person (research question 1). Second, we performed an exploratory 10×10 topic model for the #JUUL and @JUULVapor corpora and qualitatively compared differences between them. Lastly, we performed an iterative analysis to identify the optimal number of topics and again qualitatively reviewed the topic model for each corpus for differences (research questions 2 and 3).

### Ethical Use of Data

All procedures and analyses undertaken in this study conform to the Twitter’s terms for data use agreement. Our study was exempt from institutional review board review, given the secondary nature of this data collection and analysis.

## Results

### Descriptive Differences

We identified differences in total retweets and favorites per corpus. On average, content in the @JUULVapor corpus, JUUL Lab Inc’s official Twitter handle, was retweeted 1.29 times (SD 16.77 times) and favorited 0.25 times (SD 4.49 times). For the #JUUL corpus, tweets were on average favorited 0.41 times (SD 4.74 times) and retweeted 4.53 times (SD 52.07 times). Exactly 237 tweets in the #JUUL corpus originated from verified twitter accounts. Given that such a marginal number of tweets originated from a verified account, we did not perform statistical tests to determine whether scope and reach were significantly different between verified and nonverified accounts.

### Exploratory Topic Models

Table 1 outlines an exploratory topic model for the @JUULVapor corpus. This topic model represents a condensed version of JUUL Lab Inc’s 3200 most recent tweets delineated by 10 topics and 10 words per topic. The themes in the @JUULVapor’s topic model were generally interpretable. Five of the topics in the @JUULVapor topic model contained references related to customer support or product warrant-related queries—which we interpreted as responses to complaints about JUUL associated products. Words recurrent among this body of topics include *please*, *dm* [direct message], *sorry*, *thank*, *contact*, and *customer* for support-oriented topics; and *JUUL*, *device*, *limited*, *warranty*, *information* for warranty-related topics. This model also referenced adult product use (ie, *legal*, *adults*, and *age*) and acknowledgement of underage use and underage use prevention (ie, *underage*, *prevention*, *minors*, *market*, and *seriously*)—which we interpreted as JUUL Lab Inc’s forthrightly attempt to address controversies long associated with its brand. Notably, there were very few references to controversial topics such as flavors, addiction, and other drugs such as cannabis—which we interpreted as JUUL Lab Inc’s seeming attempt to distance itself from controversial aspects also associated with its brand. Other topics that emerged in this model including “Recycling” and “Warranty.” Recycling-associated tweets generally referenced the importance of recycling used JUUL cartridges (which are disposable). We interpreted warranty as a topic related to customer support—namely ways in which customers can secure refunds if products are defective.

Table 2 outlines an exploratory topic model for the #JUUL corpus, which is a random collection of tweets discussing JUUL but not originating from JUUL’s official Twitter timeline. This topic model represents a condensed version of a months’ worth of tweets about JUUL, which were identified by either using #JUUL or containing the word “JUUL” in each tweet’s text. The themes in the #JUUL topic model were somewhat interpretable, though less so than the @JUULVapor corpus reflecting greater content diversity. For example, topics that were somewhat vague, yet still referenced vaping, were labeled as a “General Vape” topic, which comprised seemingly unrelated words related to various aspects of vaping but not necessarily related to JUUL as a brand. Words recurrent among these topics include *vape*, *JUUL*, *hit*, *smoke*, *smoking*, *take*, and others. Beyond vague references to vaping, several clearer topics also emerged; these include a topic about marijuana and cannabis, the intersection of vaping and cigarettes, and a topic about nicotine, which we collectively interpreted as youth-appealing narratives about vaping. Note, topics consisting of “nicotine” or “flavors” are entirely absent from the @JUUL 10×10 topic model, which may be indicative of JUUL Lab Inc’s attempt to distance itself from web-based controversies and present a cleaner image.

**Table 1:** A 10×10 topic model of JUUL Lab Inc’s official Twitter timeline (n=3200 tweets).

Product warranty	Customer support	Purchase	Customer support	Customer support	Smoking		Underage use	Unclear		Recycle		Warranty
always	please	hi	team	please	adult	Use		hear	reaching		JUUL	
ready	dm	JUUL	know	team	JUUL	Underage		switch	thanks		device	
year	hi	JUUL Pod	working	care	smokers	JUUL		hi	currently		limited	
limited	hey	age	definitely	sorry	labels	take		JUUL	products		year	
warranty	assist	price	product	hear	lives	prevention		making	program		hey	
hi	number	retail	want	help	world	seriously		hey	team		warranty	
customer	lock	legal	frustrating	customer	billion	hi		helped	place		access	
hey	information	packs	must	contact	improving	minors		thanks	always		products	
replace	case	adults	thanks	hi	mission	product		congrats	recycle		hi	
customer	additional	available	thank	hey	high	market		leaking	quality		submit	

**Table 2:** A 10×10 topic model of a collection of tweets referencing JUUL Lab Inc and vaping. Redacted tweets refer to specific mentions which cannot be published per Twitter’s data use agreement.

General vape	Marijuana	General vape	Cigarettes	Unclear	Flavor	Nicotine	General vape	General vape	General vape
vape	vape	vape	vape	vape	JUUL	vape	vape	vape	vape
pen	cbd	JUUL	lung	JUUL	pods	vaping	vaping	police	JUUL
kid	cannabis	hit	start	mom	pod	nicotine	ublo	cops	though
city	mg	time	use	actually	hit	smoking	lungs	vaping	smoke
police	juice	think	help	two	vape	JUUL	covid	f*****g	smoking
ocean	weed	s**t	cigarettes	care	mango	shop	ml	people	take
cops	edibles	smoke	enough	vaping	days	tobacco	already	smoke	hookah
REDACTED	cbd oil	pen	cause	baby	lost	cigarettes	ecig	white	ur
vaping	liquid	f**k	says	freaking	mint	quit	vapehop	want	free
take	thc	cana	safe	stop	spring	lungs	vapelife	smoking	oh

### Iterative Topic Model

**Figure 2:**
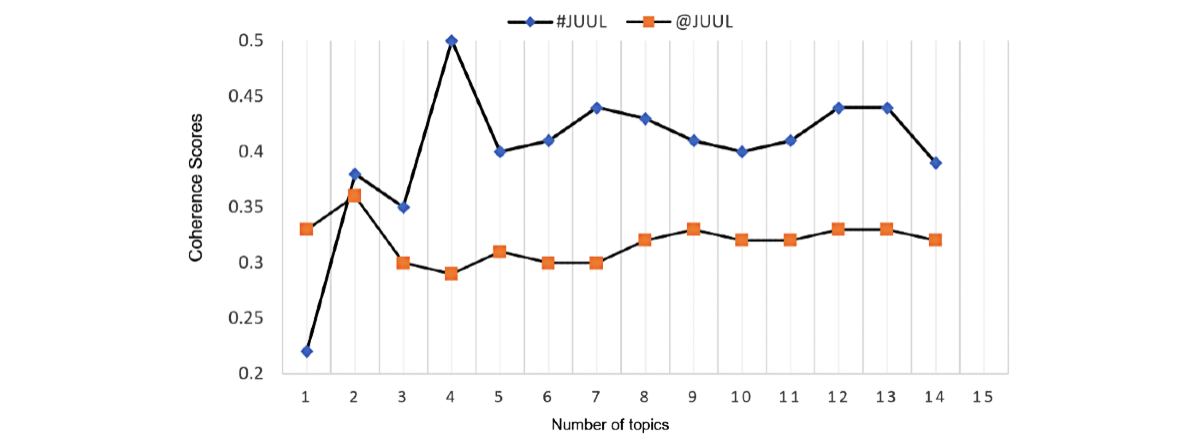
Coherence score plot by corpus. The X axis represents the total number of topics; the Y axis represents coherence score per iteration.

Iterative analysis revealed the optimal number of topics given the total number of words in each corpus. Figure 2 plots the coherence score, which measures the semantic similarity of words in each topic, per corpus [17]. Peaks in the graphs denote the optimal number of topics for each corpus.

For the @JUULVapor corpus, there were 2 optimal topics (coherence score=0.36) (Textbox 1). Both topics were interpreted as referring to either responses to customer’s concerns or complaints about JUUL products. Topics from the general topic model that centered on underage use, purchasing, and recycling were absent. This may suggest, at least partially, that the renewed purpose of JUUL Lab Inc’s official Twitter account is to field customer complaints and comments.

For the #JUUL corpus, there were 4 optimal topics (Coherence score=0.50) (Textbox 2). These topics were more diverse than the @JUULVapor corpus; containing topics related to marijuana, vape/smoking, general vaping, and vaping-related damage. Here, there is more emphasis on the elicit side of vaping/smoking, and youth appealing narratives. These topics stand in sharp contrast with the #JUUL Optimal Topic Model (Textbox 1), which only revealed customer support–related topics.

**Topics sharply contrasting the #JUUL Optimal Topic Model (coherence score=0.36). The topics in bold represent the theme; bulleted words represent the words per topic.**

**Customer complaints**

- JUUL
- hi
- use
- hey
- frustrating
- products
- must
- products
- working
- definitely

**Customer support**

- please
- team
- sorry
- hear
- care
- hi
- hey
- customer
- help
- contact

**Iterative topic model for #JUUL (coherence score=0.5). The topics in bold represent the theme; bulleted words represent the words per topic.**

**Marijuana**

- vape
- cbd
- juice
- vaping
- pen
- vapejuice
- shope
- vapelife
- weed
- cannabis

**General vape**

- vape
- vaping
- people
- smoke
- pen
- new
- lungs
- covid
- already
- day

**Vaping/smoking**

- JUUL
- vape
- pods
- hit
- nicotine
- smoke
- back
- day
- think
- hitting
- mango

**Vaping damage**

- vape
- use
- lungs
- cigarettes
- cause
- enough
- safe
- damage
- irreversible
- vanishvaping
- clear

## Discussion

### Principal Findings

This study examined the use of hashtags to indirectly market age-restricted products on social media. We leveraged the Twitter API to archive and compare 2 corpora specific to e-cigarette use with LDA topic models. The first corpus (ie, @JUULVapor) contained 3200 tweets derived from JUUL Lab Inc’s official Twitter account. The second corpus (ie, #JUUL) contained a month’s worth of tweets (May 1 to June 1, 2021) that contained #JUUL or mentioned JUUL within the tweet text (n=28,989). When the corpora were compared, we identified several telling observations within each corpus, which showcase disparate uses in @JUULVapor vs #JUUL. These partially include the @JUULVapor corpus seeming compliance to prevent underage marketing versus an array of random, youth-appealing content in the #JUUL corpus. Below we discuss these differences within the scope of their current literature delineated by each research question.

### RQ1: Evidence of Greater Reach in the #JUUL Corpus

Our first research question asked whether content about JUUL contained evidence of greater reach and visibility than content posted on JUUL’s official Twitter account. Reach and visibility, here, was measured by the average number of likes and retweets per tweet in each corpus. Our findings suggest that, overall, content about JUUL, and e-cigarettes more broadly, are clearly visible on social media spaces via hashtags. This finding corroborates a large body of work that suggests hashtags are often used to quickly distribute branding and product marketing information [4,18,19].

Regarding content, tweets in the #JUUL corpus were, on average, retweeted and liked with greater frequency than content posted by @JUULVapor. That content in the #JUUL corpus was retweeted more often than @JUUL is perhaps not entirely surprising. As mentioned previously (and throughout the remainder of the discussion), content in the #JUUL corpus contained topics of discussion that are inherently appealing to youth, versus content in the @JUUL corpus that seemed to unilaterally focus on customer complaints. For example, in the #JUUL corpus, we observed mentions of flavors (ie, mint, mango, and cucumber), cannabis vaping (ie, vape cartridges), and meme/joke-sharing, all of which are inherently conducive appealing to youth and higher post engagement. Additionally, as hashtags are used for rapid content organization of content, it is likely any social media user (agnostic of age differences) can see content posted by #JUUL, including those who did not expressly seek this information themselves.

### RQ2: LDA Topic Models as Tools to Contrast Corporate Corpora With an Assortment of Related Tweets

Beyond the reach and scope of tweets, we also investigated whether LDA could be leveraged to identify content differences in corporate versus lay user social media accounts. LDA topic models have been historically leveraged in an exploratory capacity to consolidate an overwhelming amount of text data into manageable chunks (ie, themes) that represent the most salient components of that text data [20,21]. For example, prior studies have used topic models to explore the underlying thematic structures across a broad range of corpora, including studying discourse about societal events [13], identifying alcohol branding strategies [14], and mapping publication histories of leading Health journals [22]. As topic models become increasingly used in the social and medical sciences, it remains debatable how these models can be used to test applied, rather than exploratory, hypotheses [22]. This includes ample discussion how topic models could theoretically be used to inform possible digital e-health interventions [23,24] and to construct bots from topic modeling data that meaningfully identify mental health distress [25].

To our knowledge, LDA topic models have not been used in either exploratory or applied capacities to compare social media content originating from a specific corporation and a collection of tweets about that product (though not necessarily originating from the corporate account). Our findings show that such models can be leveraged for this purpose, evidenced by our findings that identified qualitative differences in content between the @JUULVaporVapor and #JUUL corpora. We used exploratory models as a standardized metric to generate the same number of topics and words per topic for each corpus. We then ran iterative models to identify the optimal number of topics within each corpus (ie, improve granularity and precision of the models). Gethers and Poshyvanyk [26] provide more insight into granularity and relational topic models.

We contend the combined use of exploratory and iterative model may provide a conceptual framework for future topic modeling studies. For example, exploratory models may uncover broad themes in a corpus. Iterative models will then only identify highly salient (or themes of highest priority) given a corpus. The range of topics uncovered by the iterative models may highlight how broad or narrow the corpus is in scope—more themes equate to broad content in a corpus, few themes indicate narrow scope or focus. For our study @JUULVapor’s two optimal topics, contrasted with four in the #JUUL corpus, suggests the content in the @JUULVapor corpus was much narrower and more defined; for @JUULVApor, that is customer support. More topics in the #JUUL corpus suggest the content was more diverse, containing a wider array of underlying themes; that is, more youth-appealing narrative. More research is needed to identify optimal use of exploratory and precision topic models in a research context. However, we encourage the use of both exploratory and iterative models when comparing corpora of vastly different sizes.

### RQ3: Implications for Content differences between @JUULVapor and #JUUL

Our final research question posited whether content differences identified between corpora were meaningful. Across each analysis, we identified differences that clearly distinguished each corpus, including vastly different ways in which e-cigarettes were mentioned and discussed between @JUULVapor and #JUUL. This includes, as mentioned, a narrow scope of content in the @JUULVapor corpus, versus more diverse, often youth-appealing content in the #JUUL corpus. This finding, coupled with increased engagement in the #JUUL corpus supports extant research that hashtags are effective means of disseminating age-inappropriate content rapidly [3,27]. Nonetheless, deeper insights into topic nuance are needed.

First, cursory insights into JUUL Lab Inc’s corporate Twitter account show a seeming attempt to comply with FDA regulations barring youth marketing. In 2018, JUUL Labs Inc had been accused of using corporate social media accounts to market to youth and, in compliance with court orders and regulations, scrubbed their social media histories of youth-appealing content. Our inability to collect *any* deleted tweets suggests a natural limitation to social media research; namely that deleted content is truly removed from archives and cannot be accessed. However, remaining tweets posted by @JUULVapor—that is, those analyzed in this study—showcase a semiactive Twitter account almost entirely devoid of marketing content. Indeed, both exploratory and iterative topic models, the majority of topics and words per topic for @JUULVapor were customer support oriented. A review of individual tweets further revealed that the majority of posts were corporate response to complaints about JUUL products (eg, TWEET *Hi there, we’re sorry to hear that. You can access troubleshooting tips for your JUUL device at*…). This shift in content may indicate that JUUL is trying to position itself as a responsible company, similar to the corporate responsibility advertising campaigns used by Big Tobacco companies to present a respectable image while selling a dangerous product (eg, TWEET *Minors should not use any nicotine product and we take the prevention of underage use of JUUL very seriously*) [28].

By contrast, themes in the #JUUL exploratory and iterative models were more diverse and contained several references that may be appealing to youth. For example, the #JUUL corpus contained references to cartridge flavors, which have been banned in the United States because they are attractive to youth but are still legal in disposable JUUL-like products [29]. Although the JUUL company is no longer actively promoting flavors, it appears users continue to associate the JUUL product with flavors, including mango, cucumber, mint, and others. Beyond flavors, we also observed a high co-occurrence of flavors with “marijuana.” Marijuana was prominent in the exploratory #JUUL model (ie, topic 2) and retained its prominence during the iterative model. This suggests a significant portion of the #JUUL corpus contained references to cannabis. Interestingly, few tweets or topics directly mentioned JUUL (the company). JUUL not being expressly mentioned topics indicates few tweets expressly mentioned JUUL and marijuana together in the same post. However, despite not mentioning the brand directly, the web-based conversation regularly discussed the use of vape products for marijuana, which may be at least partially explained by JUUL’s evolution in mainstream vernacular form a noun (ie, JUUL products) to a verb (ie, JUUL’ing, a specific and colloquial term for “vaping”). We also observed profanity in the #JUUL topic models, which was entirely absent in @JUULVapor (eg, TWEET *Bro, we are all [expletive] high on this vape*). Profanity may indicate the presence of younger social media users [30].

Although the #JUUL corpus contained youth-appealing content (ie, profanity, high mentions of marijuana, and flavors among other indicators), we also observed topics in both @JUULVapor and #JUUL corpora detailing antivaping-related advocacy. For the @JUULVapor corpus, perhaps unsurprisingly, this may provide evidence that JUUL Labs Inc complied with court orders to stop marketing to underage users (eg, TWEET *today we’re implementing a series of new measures that build upon or existing efforts to reduce underage use*). For the #JUUL corpus, this may also suggest a substantive body of antivaping-related advocacy that adopted a hashtag strategy to spread their messages more effectively (eg, #vanishvaping). However, evidence of antivape advocacy in either corpus does not suggest that the messaging effectively deters youth use or substantively changes the wider web-based conversation. Rather, in some cases, there seem to be additional sarcastic comments that offset antivape messaging (eg, TWEET *All these anti-vape adds make me want to snort meth*)*.* Thus, such policies and court orders are ineffective in regulating the totality of messages received by underage users, particularly given that nonofficial tweets were more likely to be shared/favorited than official @JUULVapor messages. Antimessaging campaigns of other age-restricting products have also shown to have wide reach but inconclusive results [31], which may suggest that despite the best efforts of antivape advocates, their behavior change attempts may fail.

### Implications for Social Media Messaging About Vaping

Together, these findings further demonstrate the overall lack of control the official @JUULVaporVapor account has in directing web-based conversations about vape products. Despite JUUL presenting a “clean image,” their brand remains associated with a dangerous and addictive product that is naturally appealing to youth. However, it is also clear that more research on who is tweeting about JUUL and vaping, and how hashtags facilitate marketing illicit behavior, is needed. Future research should consider adding deep learning models to partition tweets about vaping by demographic variables to, among other matters, predict the likelihood an account posted about JUUL was that of an underage user.

From a public health/medical/interventionist perspective, our findings also compel us to ponder how mining communication patterns (ie, tracking discourse about JUUL) can be further leveraged to identify intervention targets promoting antivape messaging. In this study, the sharp divide in content between @JUUL and #JUUL suggest that provape messaging did not end after JUUL Lab Inc’s court order to curb marketing efforts. Rather, marketing manifested through shared user content about products that remain popular while not necessarily referencing the elicit product. It is indeed possible that some influencers are paid by manufacturers such as JUUL to surreptitiously market products without seeming association with the brand. This was a strategy used by Big Tobacco to continue marketing indirectly while seemingly complying with antimarking efforts. Additionally, on Twitter, it is difficult to determine which posts from celebrity and other verified accounts are paid advertisements. Other platforms, including Instagram and Facebook, expressly designate ads with a “#ad” notice; however, this is less common with Twitter. Policy efforts should also center on clearer guidelines for designating paid or sponsored posts versus regular posts on Twitter.

### Limitations

Our study is subject to limitations we hope to address in future work. First, we acknowledge a likely demographic bias inherent to social media studies. This includes a sample that likely skews younger, male, wealthier, and whiter than the general population [32]. Given that our study was exploratory, we also did not control spatial and geographic patterns in social media data, which affect how and what users post within a given time (ie, rural vs urban settings, or older users posting earlier in the morning than younger users) [33,34]. Regarding data analysis, we acknowledge that we did not perform a formal qualitative analysis with these data. Topic models were used to, instead, consolidate each corpus and allow us to draw inferences about the data from those topics. Because of sample size constraints, we were also unable to draw meaningful comparisons between verified and nonverified users in the #JUUL corpus. However, despite these limitations, we believe gaps in our study present opportunities for future research related to social media discourse on age restricted products. This includes performing other NLP methodologies with similar data (ie, sentiment analysis) to understand polarity in discourse or applying classifiers to accounts tweeting about age-restricted products to predict age and gender among other demographic traits. Dai et al [35] have provided further information about the M3 classifier in a health context. Such studies may provide a deeper and more nuanced landscape of social media discourse related to age-restricted products.

### Conclusions

@JUUVapor may be compliant with web-based marketing restrictions and promoting antivaping messaging. However, JUUL Labs Inc is powerless to control the larger narrative about vaping on social media. Indeed, hashtags about vaping and JUUL contain much of the youth-directed content that led to the initial impositions placed on JUUL by the FDA. Our results underscore the difficulty of regulating social media content despite federal impositions that ban marketing of age-restricted products in web-based spaces. Given the limited ability of social media to restrict what underage users see on their websites, companies can bypass marketing regulations by allowing users to freely share related hashtags or by paying social media influencers to disseminate these hashtags [36]. Although it is impossible to regulate free speech on the internet, perhaps public health advocates could harness the power of hashtags to deliver antivaping messages, though the effectiveness of such campaigns is not guaranteed.

## Abbreviations

API: application programming interface
FDA: Food and Drug Administration
LDA: Latent Dirichlet Allocation
NLP: natural language processing
